# Rooted in Joy: Exploring Sources of Joy Among Older African American Women

**DOI:** 10.1093/geroni/igaf122.3650

**Published:** 2025-12-31

**Authors:** Jacquelyn Coats, Sheltoria Love, Anais Mahone

**Affiliations:** Duke University School of Medicine, Durham, North Carolina, United States; Washington University in St. Louis, St. Louis, Missouri, United States; University of Alabama at Birmingham, Birmingham, Alabama, United States

## Abstract

Older African American women demonstrate remarkable resilience despite persistent stressors and disproportionate health outcomes compared to other groups. While joy has been identified as a potential contributor to health and well-being, it remains understudied among this population. This qualitative study addresses that gap by exploring sources of joy in later life for this important group. The analysis is nested within a larger constructivist grounded theory project that used semi-structured interviews to examine stress and coping among college-educated African American women. This study focuses on responses to the final interview question: “What brings you joy?” Participants reflected on joy within the context of their broader life experiences. Sixteen women aged 65 to 91 were recruited through community organizations, libraries, and social networks in the Midwest. Guided by Black Feminist Theory, a collaborative, discussion-based thematic analysis was used to interpret the data. Narratives revealed diverse and multidimensional sources of joy. Four overarching themes emerged: (a) experiencing and preserving Black culture, (b) play and adventure, (c) spirituality and life purpose, and (d) community and connection. Findings suggest that joy is a vital and culturally grounded source of resilience and well-being for older African American women. For aging and gerontology researchers and health practitioners, understanding and integrating elements that foster joy may enhance culturally responsive care and support. This study also underscores the need for focused research on joy as a distinct phenomenon shaped by cultural and structural contexts, and its potential role in promoting health equity in later life.

